# Two cases of pararenal artery aortic aneurysm treatment after pancreaticoduodenectomy and abdominal aortic aneurysm stent grafting

**DOI:** 10.1186/s40792-024-01834-9

**Published:** 2024-02-09

**Authors:** Kazuhiro Yamazaki, Kenji Minatoya, Kazuhisa Sakamoto, Masafumi Kudo, Ken Fukumitsu, Takashi Kobayashi, Hideaki Okajima

**Affiliations:** 1 Division of Cardiovascular Surgery, Department of Surgery, Shimane University Faculty of Medicine, Izumo, Shimane Japan; 2 Department of Cardiovascular Surgery, Graduate School of Medicine, Kyoto University, Kyoto, Japan; 3 Department of Surgery, Graduate School of Medicine, Kyoto University, Kyoto, Japan; 4 Department of Urology, Graduate School of Medicine, Kyoto University, Kyoto, Japan; 5 Department of Pediatric Surgery, Kanazawa Medical University, Kanazawa, Japan

**Keywords:** Pararenal artery aortic aneurysm, Pancreaticoduodenectomy, Acute pancreatitis, Autologous kidney transplantation, Endovascular aneurysm repair

## Abstract

**Background:**

Acute pancreatitis caused by surgical procedures may occur less frequently in surgeries for aortic aneurysm involving the abdominal branch. However, in such cases, the associated mortality rate increases significantly. There have been few reports on abdominal aortic aneurysm surgery after pancreatoduodenectomy; as such the incidence of postoperative pancreatitis remains unclear.

**Case presentation:**

Two cases of pararenal artery aortic aneurysm after pancreaticoduodenectomy and endovascular aneurysm repair (EVAR) for an abdominal aortic aneurysm are reported. In the first case, a 74-year-old man was diagnosed with abdominal aortic aneurysm and duodenal cancer 6 years earlier and underwent pancreaticoduodenectomy after EVAR. Subsequently, the abdominal aorta expanded to 58 mm at the level of the renal artery proximal to the EVAR site. Graft replacement was performed through a left thoraco-retroperitoneal incision. However, the patient died from acute pancreatitis, believed to be caused by intraoperative manipulation. Given this initial experience, in the second case, a 77-year-old man had undergone a pancreaticoduodenectomy for a gastrointestinal stromal tumor 17 years earlier and EVAR for an abdominal aortic aneurysm 10 years earlier. The abdominal aorta had expanded to 50 mm immediately below the right renal artery on the proximal side of the EVAR. Subsequently, hematuria was noted, and he was diagnosed with right ureteral cancer. Autologous transplantation of the left kidney and EVAR was performed avoiding manipulation of the area around the pancreas and achieved good results. Combined right renal and ureteral resections were performed 20 days after EVAR.

**Conclusions:**

While performing aortic surgery after pancreaticoduodenectomy, surgeons should avoid manipulating tissues around the pancreas.

## Background

Gastrointestinal complications may occur in surgeries for aortic aneurysm involving the abdominal branch due to the surgical procedure and the influence of blood flow in the abdominal branch [1]. Similarly, acute pancreatitis can occur after surgery. Its incidence is not high, but it increases mortality rate significantly [2, 3]. Here, we report two cases of abdominal aortic aneurysms during the chronic phase after pancreaticoduodenectomy with different courses of treatment and outcomes. In the first case, the patient died due to acute pancreatitis following artificial graft replacement. The second patient had a good outcome with endovascular aneurysm repair (EVAR) after autologous kidney transplantation.

## Case presentation

### Case 1

**Fig. 1:**
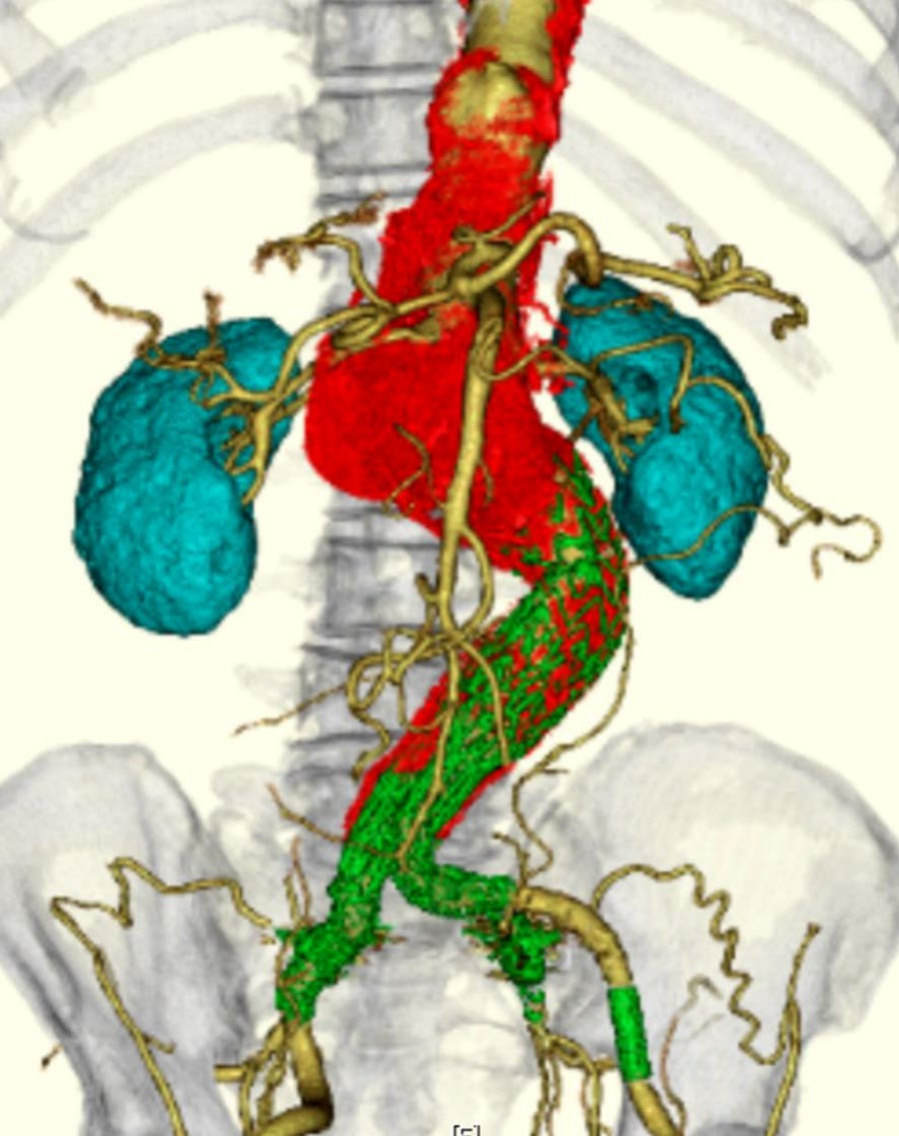
Preoperative 3D computed tomography scan of the first patient

**Fig. 2:**
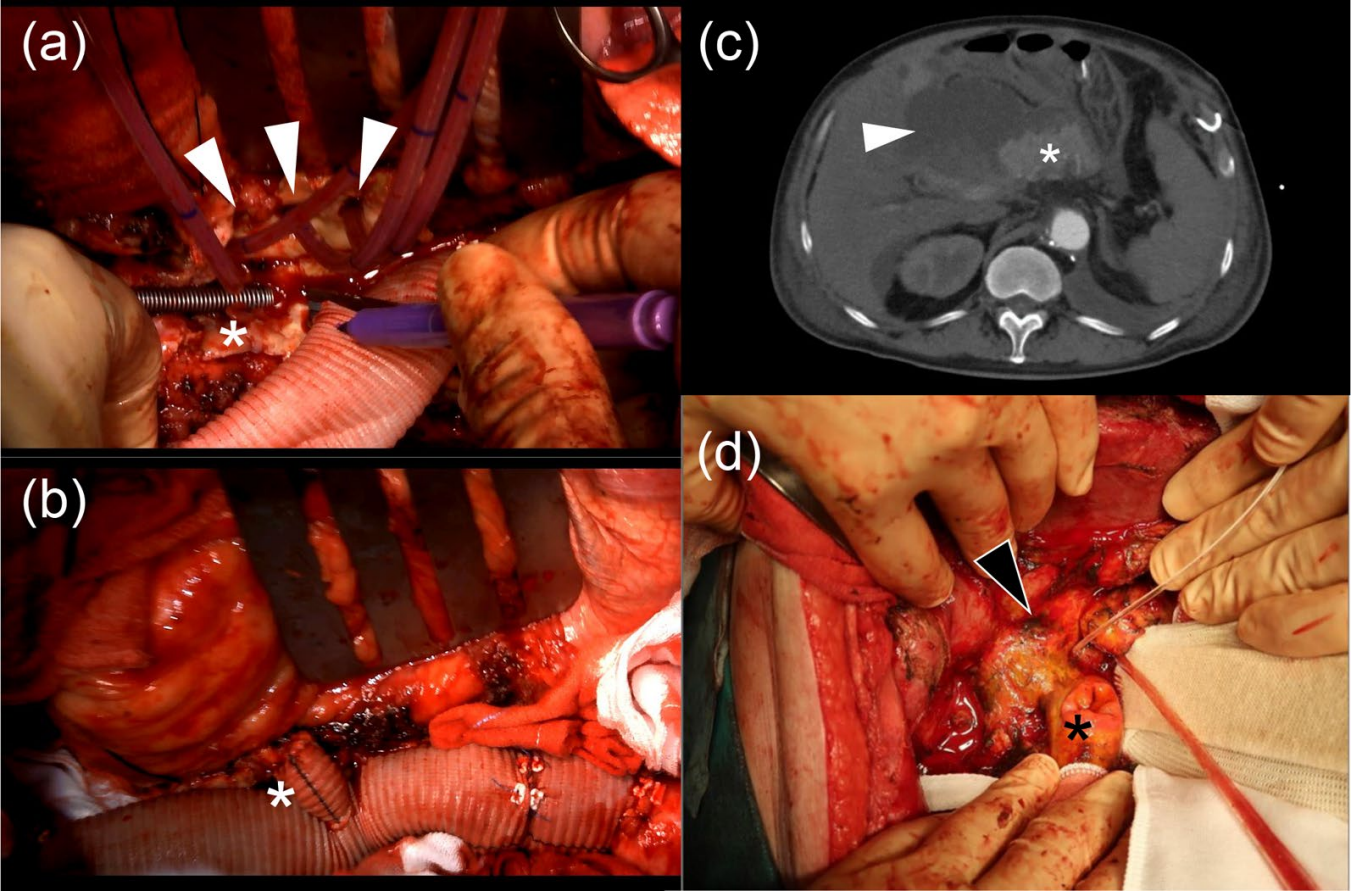
Operative findings during the expansion of the surgical field around the pancreas. **a** The abdominal branch was selectively perfused, and its branches other than the left renal artery (asterisk) were anastomosed by island reconstruction (arrow heads). **b** The left renal artery was reconstructed using a side branch (asterisk). **c** On the 17th postoperative day, computed tomography revealed fluid retention (arrow) around the pancreas (asterisk). **d** Emergency surgery findings: the pancreaticoduodenal anastomosis had completely disappeared (arrow: pancreatic duct tube and anastomosis; asterisk: anastomosis of duodenal side of the anastomosis)

A 74-year-old man had been diagnosed with abdominal aortic aneurysm and duodenal cancer 6 years earlier and underwent pancreaticoduodenectomy after EVAR. Subsequently, the abdominal aorta expanded to 58 mm at the level of the renal artery proximal to the EVAR site (Fig. 1), and the patient was referred to our hospital. Due to adhesions around the pancreas, access through a midline abdominal incision was considered difficult. Therefore, we selected a retroperitoneal approach for graft replacement using the cardiopulmonary bypass. The aneurysm was exposed thoraco-retroperitoneal incision in the sixth intercostal space. After establishing partial cardiopulmonary bypass, the aorta was clamped peripherally and centrally, and the aneurysm was incised. The abdominal branches were selectively perfused (Fig. 2a). First, a central anastomosis was performed using a 24-mm single-branch Gelweave graft (Terumo, Tokyo, Japan). After the stent graft was removed, its branches other than the left renal artery were anastomosed by island reconstruction, and peripheral anastomosis was performed. Finally, the left renal artery was reconstructed using a side branch of the graft (Fig. 2b). Blood examination on postoperative day (POD) 1 revealed high serum levels of pancreatic enzymes (amylase, 1868 U/L; lipase, 1468 U/L). On POD 17, muscular guarding appeared, and fluid retention around the pancreas was detected by computed tomography (Fig. 2c). Hence, computed tomography-guided percutaneous transhepatic drainage was performed. The drained fluid was dark red, and its amylase level was extremely high (21,486 U/L); therefore, the patient was diagnosed with a pancreatic fistula. Two days later, emergency surgery was performed for intestinal perforation. Intraoperative findings showed that the pancreaticoduodenal anastomosis had completely disappeared (Fig. 2d). He subsequently developed gastrointestinal perforations and died on POD 135 due to multiple organ failure.

### Case 2

**Fig. 3:**
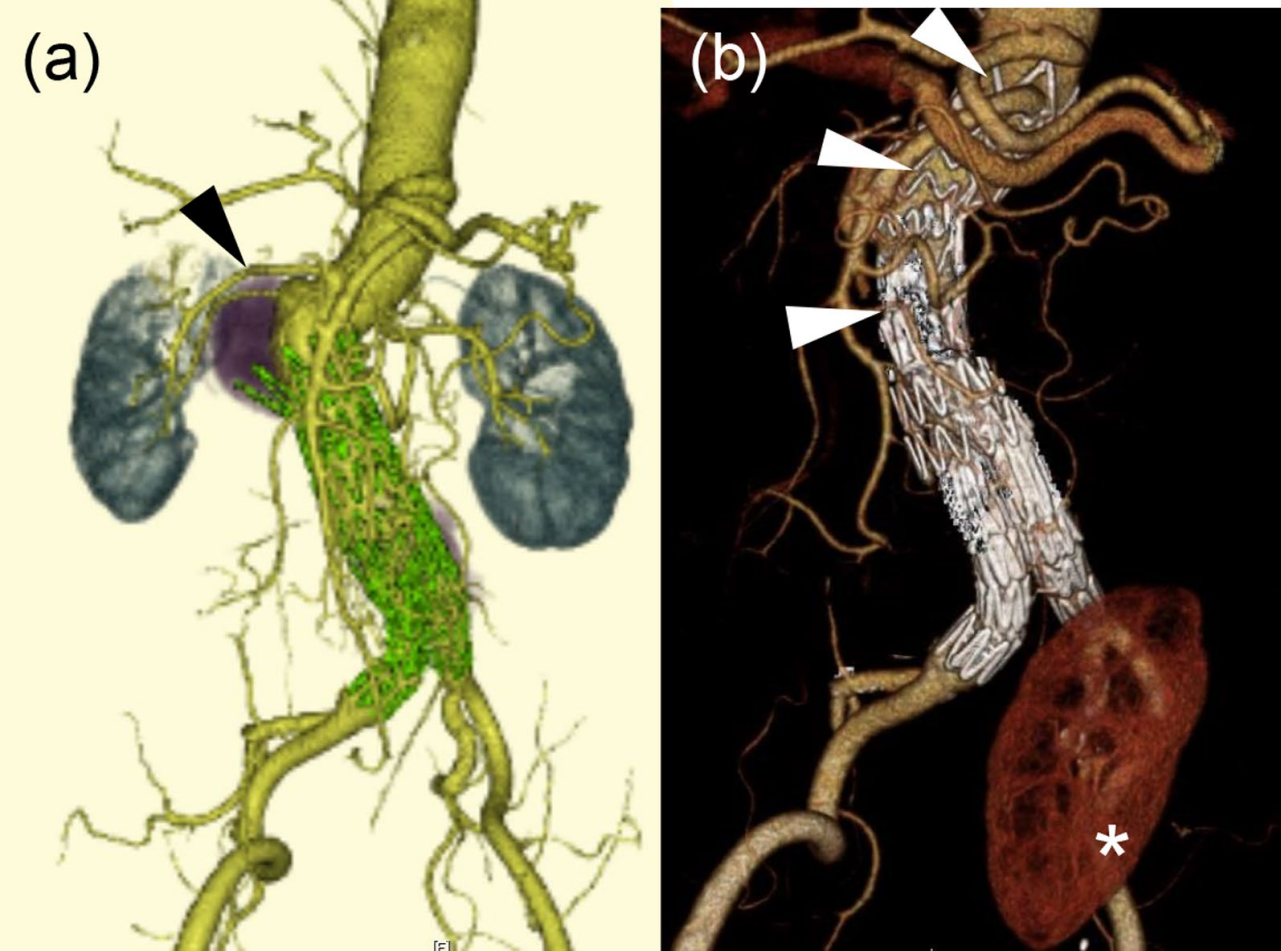
**a** Preoperative computed tomography scan of the second case. The abdominal aorta has expanded to 50 mm in size, just below the right renal artery (arrow heads) on the proximal side of the EVAR. **b** Postoperative computed tomography-scan of the second patient. EVAR was performed by extending the proximal side of the previous stent-graft (arrow heads) and the autologous left kidney was transplanted (asterisk)

A 77-year-old man had undergone a pancreaticoduodenectomy for a gastrointestinal stromal tumor (GIST) 17 years earlier and an EVAR for an abdominal aortic aneurysm 10 years earlier. The GIST had recurred 2 years earlier, and conservative treatment was initiated. The abdominal aorta had expanded to 50 mm immediately below the right renal artery on the proximal side of the EVAR (Fig. 3a). Subsequently, hematuria was noted, and he was diagnosed with right ureteral cancer. Therefore, he was referred to the urology department of our hospital, and a combined resection of the right kidney and ureter was planned. Because the right renal artery originated from the aneurysm and protruded to the right, we judged that performing the urological surgery with the aneurysm would be dangerous; hence, we decided to treat the aneurysm before the urological procedure. In this case, as in Case 1, an approach through a midline abdominal incision and aortic clamping around the visceral arteries were considered difficult due to adhesions around the pancreas. Moreover, additional EVAR was likely to block blood flow to the left renal artery. Therefore, autologous left kidney transplantation was first performed to obtain a safe landing zone to perform EVAR. A stent graft was then placed by extending the proximal side of the previous stent graft without causing deterioration of renal function on day 19 after the transplantation. The combined right renal and ureteral resections were performed 20 days after the EVAR (Fig. 3b). The patient has been in good condition for 2 years after these procedures.

## Discussion

The incidence of pancreatitis after open-heart surgery is 0.4–6%. However, this varies due to differences in the diagnostic criteria for pancreatitis [2–4]. Pancreatitis after abdominal aortic aneurysm surgery occurs at almost the same rate [5, 6]. However, when it occurs after abdominal aortic aneurysm surgery, it is associated with severity and mortality rates that range from 10 to 50% [2–6]. A decrease in the pancreatic blood flow can cause pancreatitis after surgery [1, 2].

Pancreatitis is associated with an increase in serum amylase and lipase levels. In general, serum amylase levels often show a bimodal transition, with a first peak of salivary gland isozyme dominance immediately after surgery and a second peak of pancreatic-type isozyme dominance approximately 3–5 days after surgery [7–9]. Furthermore, it has been reported that the second peak coincides with the peak in lipase levels [7]. In our first case, amylase and lipase levels increased the day after the operation. Therefore, we believe that the diagnosis of postoperative pancreatitis was appropriate. However, it was different from the above-mentioned pattern of the onset of pancreatitis after open-heart surgery. This difference is believed to be due to the preoperative history of pancreaticoduodenectomy. The adhesions after the pancreaticoduodenectomy might have caused excessive stress around the pancreatic anastomosis when the surgical field around the pancreas was being expanded during surgery. In fact, the laparotomy findings showed that the pancreatic duct anastomosis had completely disappeared (Fig. 2d). Akabane et al. reported a case of postoperative pancreatitis in which pancreatic amylase levels increased the day after surgery for a thoracoabdominal aortic aneurysm in the chronic phase following a pancreaticoduodenectomy [10]. These findings suggest that aortic aneurysm repair via a Stoney incision after pancreaticoduodenectomy should be performed with serious caution.

Based on these data, a surgical method for avoiding detachment around the pancreas was considered in the second case. We investigated methods to eliminate the risk of bleeding from the aortic aneurysm in order to safely perform a combined resection of the right kidney and ureter. Therefore, EVAR was our first choice. However, the need to preserve the left kidney during such a procedure required consideration. We considered the so-called chimney procedure, in which a renal artery stent is placed in the left renal artery, followed by EVAR. However, we did not choose this option because if an endoleak, especially gutter leak, were to remain, a safe nephrectomy might be impossible. Therefore, to ensure treatment of the aneurysm and eliminate the risk of aneurysm rupture, we performed EVAR with adequate landing zone after autotransplantation of the left kidney. Subsequently, the resection could be safely performed. The renal artery was not coiled but only covered with a stent graft because we believed that EVAR might affect the arterial processing during nephrectomy. Fortunately, no major complications due to renal infarction occurred after stent placement. Autologous kidney transplantation has been recently reported as a useful method for thoracoabdominal aortic aneurysm surgery [11–13].

## Conclusion

We performed aortic aneurysm surgery extending to the abdominal branch during the chronic phase after pancreaticoduodenectomy in two cases. One patient died due to acute pancreatitis, which we believe was caused by intraoperative manipulation. For aortic surgery after pancreaticoduodenectomy, a carful preoperative examination should be performed and manipulation of the tissues around the pancreas should be avoided during surgery. In addition, we believe that EVAR after autologous kidney transplantation is a useful method for treating aneurysms around the abdominal branch.

## Data Availability

Data sharing is not applicable to this article, as no datasets were generated or analyzed during the study.

## Abbreviations

EVAR: Endovascular aneurysm repair
POD: Post-operative day
GIST: Gastrointestinal stromal tumor
